# Cross-modal plasticity in the deaf enhances processing of masked stimuli in the visual modality

**DOI:** 10.1038/s41598-017-08616-4

**Published:** 2017-08-15

**Authors:** Seema Prasad, Gouri Shanker Patil, Ramesh Kumar Mishra

**Affiliations:** 1 Center for Neural and Cognitive Sciences, University of Hyderabad, Hyderabad, India; 2 Ali Yavar Jung National Institute for the Hearing Handicapped, Secunderabad, India

## Abstract

Compensatory changes as a result of auditory deprivation in the deaf lead to higher visual processing skills. In two experiments, we explored if such brain plasticity in the deaf modulates processing of masked stimuli in the visual modality. Deaf and normal-hearing participants responded to targets either voluntarily or by instruction. Masked primes related to the response were presented briefly before the targets at the center and the periphery. In Experiment 1, targets appeared only at the foveal region whereas, in Experiment 2, they appeared both at the fovea and the periphery. The deaf showed higher sensitivity to masked primes in both the experiments. They chose the primed response more often and also were faster during congruent responses compared to the normal hearing. These results suggest that neuroplasticity in the deaf modulates how they perceive and use information with reduced visibility for action selection and execution.

## Introduction

Much of what we do not see and report nevertheless influences our actions and thought. Many studies in the domain of visual perception have shown that subliminally presented primes can influence action selection and control^1–5^. Such unconscious states give rise to overt actions later^6, 7^. Although unconscious priming as an effect and as a paradigm remains debated, studies tightly controlled for prime visibility show a substantial influence of the primes on later behaviour (e.g. ref. 8). It’s not clear if variables related to individual differences in biological and cognitive profiles should influence the processing of hardly-visible stimuli. One such variable is neural reorganisation as a result of sensory deprivation as it happens in the deaf or the blind. Here, we examine if the deaf show higher sensitivity to masked priming. We exploit the deaf participants since they have been found to perceive and react faster to visual stimuli (e.g. refs 9, 10). Additionally, we also explored if the priming effects vary for the foveal and peripheral regions, as the deaf have been shown to be particular faster in discrimination at the periphery (e.g. ref. 11).

Cortical reorganisation following a lack of input to the auditory cortex has been observed in the Deaf^12, 13^. Mainly, structural and functional enhancement in the dorsal processing stream has been reported in the deaf (see ref. 14 for a review). As a result, the deaf have been shown to be more sensitive to information in the visual periphery and also display higher spatial attention particularly at the visual periphery^10, 11, 14–19^. Although many have found that the Deaf are faster with visible stimuli compared to the normal hearing, we hypothesize that they should be highly sensitive to stimuli in the environment that have reduced visibility.

The source of the predicted enhanced priming effects in the deaf may lie in the selective manner in which the dorsal stream functions^19–21^, see also^17, 22^ for reviews) The dorsal route hypothesis, as it is called, makes explicit predictions about superior spatial attentional abilities in the deaf. Visual spatial attention is redistributed more widely in the deaf extending to the periphery^13, 23–25^. For instance, Proksch and Bavelier^25^ observed that the Deaf processed peripheral distractors more while performing a search task at the center of the visual field, compared to normal hearing individuals. In consonance with the dorsal route hypothesis, ERP data (N1 component) showed preferential processing of ‘motion’ stimulus (dorsal) compared to ‘color’ stimulus (ventral) in the deaf^26^. But does such a reorganisation of the ‘dorsal’ stream lead to higher sensitivity to information that is hardly visible in the deaf?

**Figure 1:**
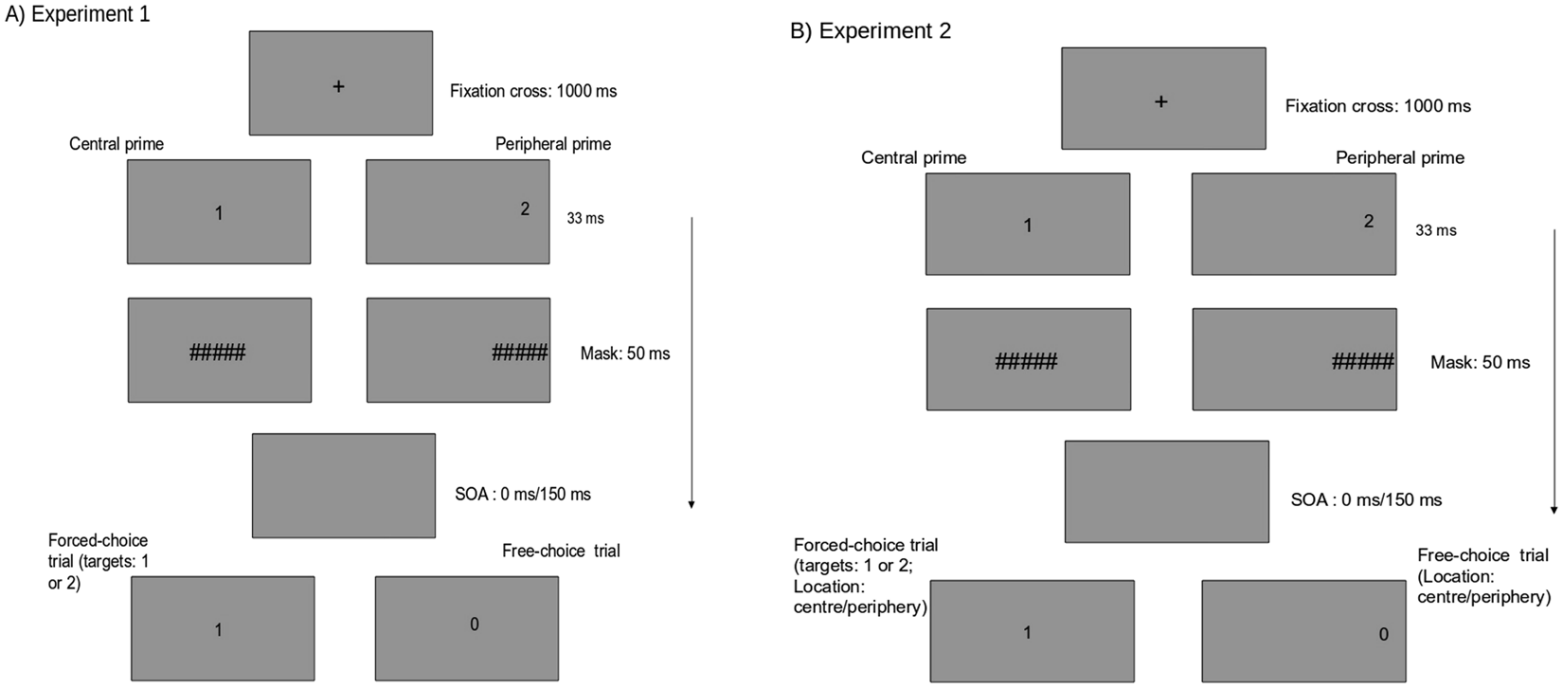
Experimental design. (**A**) Experiment 1. Primes (1 or 2) are presented at centre or periphery followed by a mask (#####). Participants then respond to the target cue (forced-choice trials) or choose between two alternative responses (free-choice trials). The target numbers (1, 2 or 0) are always presented at the centre. (**B**) Experiment 2. The design is similar to Experiment 1. The only difference is that targets also presented at periphery, always matching the prime location.

Most researchers so far have focused on the peripheral visual advantage in the deaf to perceivable visible stimuli. It is clear that the deaf show higher sensitivity to stimuli presented at the visual periphery. Although, it is not certain if this advantage is attentional or perceptual given the different paradigms and tasks researchers have used. Given this background, we explored if the deaf show higher sensitivity to masked primes which in turn would influence their action related choices? Additionally, we sought to examine if such an effect is particularly higher at the visual periphery for the deaf. It has been suggested that deaf perform better with these visual tasks if the attentional demands are high. We used masked primes to modulate free- and forced-choice visuomotor actions in the deaf and the normal hearing participants (Fig. 1). In Experiment 1, the primes were presented either at the periphery or center whereas targets appeared only at the center. In Experiment 2, the target location always matched the prime location. This was done to test whether the peripheral advantage in Deaf would still be seen when the attentional demands are low (Experiment 2).

We also manipulated the mask-target SOA based on a previous study examining central-peripheral masked priming effects^27^. In this study, masked primes were presented briefly followed by targets. Left or right pointing arrows were used as primes/targets and several of them were superimposed on each other to create the mask. Participants were asked to respond to the direction of the arrow. Crucially, the mask-target SOA was varied from 0 ms to 192 ms. A positive compatibility effect (PCE, faster responses on compatible trials) at shorter SOAs and negative compatibility effect (NCE, faster responses on incompatible trials) at longer SOAs is commonly observed when mask-target SOA is varied. The NCE is said to represent a self-inhibition mechanism which causes the suppression of the initial activation of the prime. Accordingly, in this study, PCE was observed at shorter SOAs (<96 ms) which turned into NCE at longer SOAs (>96 ms) as expected but only for central primes. For peripheral primes, PCE was observed even at longer SOAs. The explanation for this so-called central-peripheral asymmetry (CPA) was that the activation-followed-by-inhibition account is valid only when primes are presented at the fovea. At other locations, the perceptual strength of the primes is not strong enough to cross the activation threshold and result in inhibition. However, it is known that Deaf have high perceptual sensitivity even at the periphery. Thus, such an asymmetry between central and peripheral priming effects should be observed only in normal-hearing participants but not in the Deaf. In sum, we expected normal-hearing participants to display CPA at long SOA similar to previous studies^27^. However, we expected no such asymmetry in the Deaf. We expected these effects on both free- and forced-choice trials as NCE has been observed in both types of trials^36^.

## Experiment 1

The main aim of Experiment 1 was to test the hypothesis that the Deaf should show enhanced masked priming effects. Deaf and normal-hearing participants completed a masked priming task in which they were asked to make responses to targets (“forced-choice” trials) or voluntarily choose a response between two alternatives (“free-choice” trials). We expected the Deaf to show higher priming effects on both free- and forced- trials. That is, we expected the proportion of congruent choices (on free-choice trials) and RT priming effect (RT incongruent – RT congruent, on free- and forced-choice trials) to be higher in the Deaf. A prime discrimination test was administered after the main experiment to test for the visibility of the primes. Based on prior evidence^20, 28, 29^ we did not expect to see any differences between Deaf and normal-hearing in the prime visibility index (*d* prime).

### Data analysis

Free- and forced-choice trials were analysed separately (See Table 1 for descriptive statistics). Statistical analysis was performed using SPSS (IBM Corp.). F and p values along with partial eta squares are mentioned for all the relevant main effects and interactions. Pairwise comparisons on significant interactions were performed using LSD posthoc tests.

**Table 1:** Descriptive Statistics – Experiment 1.

			Central primes				**Peripheral primes**			
			**0**		**150**		**0**		**150**	
			**C**	**IC**	**C**	**IC**	**C**	**IC**	**C**	**IC**
**Free- choice**	**Choices**	**Deaf**	57 (15)	43 (15)	62 (20)	39 (20)	55 (16)	45 (16)	57 (15)	43 (15)
		**NH**	53 (9)	47 (9)	52 (9)	48 (9)	49 (8)	51 (8)	51(11)	49 (11)
	**RT**	**Deaf**	689 (168)	761 (295)	625 (137)	757 (295)	717 (254)	778 (305)	654 (148)	681 (263)
		**NH**	646 (198)	659 (187)	602 (205)	630 (227)	638 (208)	649 (210)	631 (200)	625 (191)
**Forced- choice**	**Error rate**	**Deaf**	0.6 (1)	1.2 (2)	0.6 (1)	1.9 (3)	0.9 (1)	0.9 (2)	0.7 (1)	1.1 (0.2)
		**NH**	0.5 (1)	0.8 (2)	0.5 (1)	1 (2)	0.6 (1)	0.3 (1)	0.6 (1)	10.8 (0.1)
	**RT**	**Deaf**	648 (143)	704 (182)	594 (155)	681 (181)	691 (202)	703 (200)	673 (148)	694 (182)
		**NH**	637 (168)	656 (155)	565 (136)	590 (122)	629 (159)	626 (164)	597 (148)	585 (126)

Note: Mean values with SD in brackets. C: Congruent, IC: Incongruent, NH: Normal-hearing, “0” and “150” refer to mask-target SOA in ms, Choices and error rates are given in percentage (%). RT is given in ms.

#### Forced-choice trials

Trials with no responses were discarded (0.7%). Response times on forced-choice trials above and below 2 SD (Standard deviation) of the Mean RT of each participant were also discarded (4.4% in the Deaf and 4.8% in the normal-hearing). Further, only correct trials were considered for the main analysis. The percentage of errors was 7.8% for the Deaf and 5.1% for the normal-hearing. Error analysis was performed separately by computing *d*′ values. Correct responses to the target “1” were defined as hits and incorrect responses to “2” were defined as false alarms. Hits and false alarm rates of 0 or 1 were corrected using the log-linear rule^30^. Please note that the mean values for different conditions are mentioned in percentage for the ease of comprehension.

#### Free-choice trials

In line with previous studies on free-choice priming, stringent data trimming procedure was not applied to free-choice trials (e.g. refs 27, 31. Instead, only responses faster than 100 ms (Deaf: 0.03% and Normal-hearing: 0.08%) and trials with no responses (0.8%) were discarded. We computed *d*′ values for each participant for each condition. A response was considered as a hit if the participants chose “A” when the prime was “1” and as a false alarm if the participants chose “A” when the prime was “2” (The calculation was adjusted accordingly when the mapping was opposite). ANOVA was performed on the *d*′ values. Individual t-tests comparing with chance level performance (*d*′ = 0) were also performed wherever appropriate. Means are, however, reported in percentage.

## Results

### Free-choice trials

**Figure 2:**
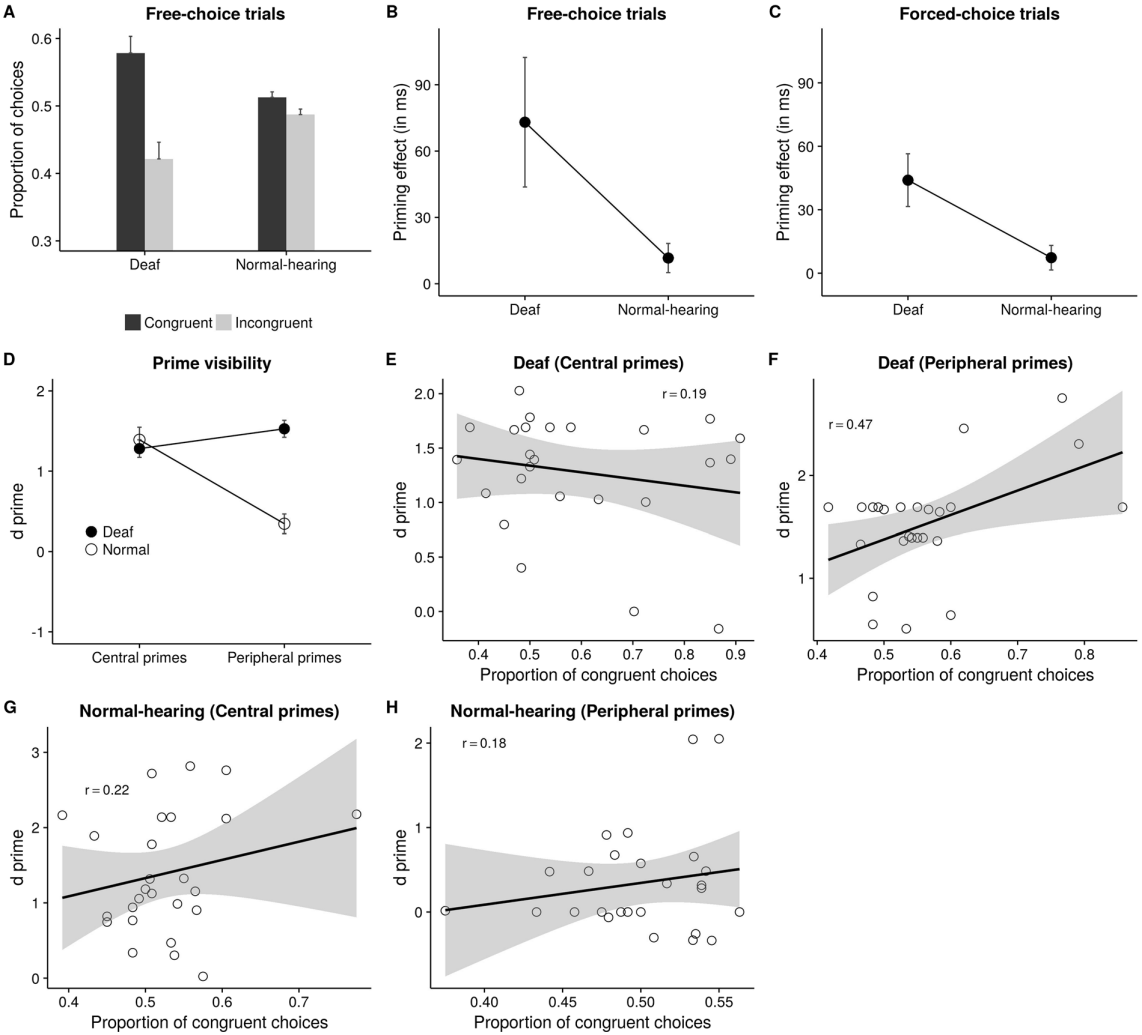
Experiment 1 results. (**A**) Deaf made more congruent choices on free-choice trials compared to normal-hearing. The RT priming effect (RT incongruent – RT congruent) is larger for the Deaf on both free-choice (**B**) and forced-choice (**C**) trials. (**D**) Deaf are better at discriminating primes, only at periphery. (**E**–**H**) Plots of correlation between proportion of congruent choices at the two prime locations for the two groups. The effect was significant (*p* = 0.02) only for the Deaf at periphery (**F**).

Congruent choices were more in number (54.6%) than incongruent choices (45.4%), *t* (1, 50) = 3.24, *p* = 0.002. ANOVA was then performed on *d*′ values of proportion data with congruency (congruent, incongruent), prime location (central, peripheral), SOA (0, 150) as within-subjects factors and group (Deaf, normal-hearing) as a between-subjects factor. There was a significant main effect of group, *F* (1, 49) = 5.78, *p* = 0.02, *η*_*p*_^2^ = 0.10. indicating that Deaf (57.9%) made higher proportion of congruent choices than normal-hearing (51.3%, Fig. 2A). Individual t tests revealed that *d*′ differed significantly from zero for the Deaf (*d*′ = 0.32), *t* (1, 25) = 3.1, *p* = 0.005 but not for normal-hearing participants (*d*′ = 0.05), *t* (1, 24) = 1.44, *p* = 0.16. There was also a main effect of prime location, *F* (1, 49) = 5.65, *p* = 0.02, *η*_*p*_^2^ = 0.10 indicating that the participants made higher proportion of congruent choices for central primes (56%) compared to peripheral primes (53.2%). The main effect of SOA [*F*(1, 49) = 1.11, *p* = 0.3, *η*_*p*_^2^ = 0.02] and all of the interactions were nonsignificant (*F*s < 1).

Primes had an influence on response times on free-choice trials, as indicated by a main effect of congruency on RT on free-choice trials, *F* (1, 49) = 8.25, *p* = 0.006, *η*_*p*_^2^ = 0.14. Congruent trials (650.18 ms ms) had faster responses than incongruent trials (692.54 ms). To further examine the effect of other variables on priming effect, ANOVA was performed on “RT priming effect” which was calculated as the RT difference between incongruent and congruent trials. Prime location, SOA and Group were considered as factors. There was a significant effect of group, *F* (1, 49) = 4.34, *p* = 0.04, *η*_*p*_^2^ = 0.08 reflecting a larger effect of the primes for the Deaf (73.07 ms) compared to the normal-hearing participants (11.64 ms, Fig. 2B). Priming effect for central primes (61.35 ms) and peripheral primes (23.36 ms) differed, as indicated by a main effect of prime location, *F*(1, 49) = 4.44, *p* = 0.04, *η* ^2^ = 0.08. The interaction between prime location and group was not significant, *F* (1, 49) = 1.12, *p* = 0.28, *η*_*p*_^2^ = 0.02. There was no main effect of SOA (*F* < 1). None of the interactions were significant (*F*s < 3).

### Forced-choice trials

Repeated measures ANOVA was first performed on RT with congruency (congruent, incongruent), prime location (central, peripheral) and SOA (0 ms, 150 ms) as within-subjects factors and Group (Deaf, normal-hearing) as between-subjects factors. There was a main effect of congruency on RT, *F*(1, 49) = 14.26, *p* < 0.001, *η*_*p*_^2^ = 0.22 indicating that participants were faster responding on congruent trials (629.16 ms) compared to incongruent trials (654.82 ms). Further analysis of RT priming effect (incongruent RT – congruent RT) revealed higher priming effect for the Deaf (44 ms) compared to normal-hearing (7.32 ms) participants, *F*(1, 49) = 7.28, *p* = 0.01, *η*_*p*_^2^ = 0.13, as shown in Fig. 2C. A main effect of prime location, *F*(1, 49) = 20.26, *p* < 0.001, *η*_*p*_^2^ = 0.29 showed larger priming effects for primes for central primes (46.65 ms) compared to peripheral primes (4.67 ms). The interaction between prime location and group was not significant, *F* (1, 49) = 1.95, *p* = 0.17. Neither the main effect of SOA [*F*(1, 49) = 1.26, *p* = 0.27, *η*_*p*_^2^ = 0.02] nor its interactions with group [*F*(1, 49) = 1.89, *p* = 1.7, *η*_*p*_^2^ = 0.04] and location [*F*(1, 49) = 1.05, *p* = 0.31, *η*_*p*_^2^ = 0.02] was significant. The interaction between prime location, SOA and Group, *F* (1, 49) = 0.01, *p* = 0.91, *η*_*p*_^2^ *< *0.001 was not significant either.

Percentage of errors on forced-choice trials (there was no concept of error on free-choice trials) was also analysed with prime location, congruency, SOA and Group as factors. The ANOVA revealed a significant effect of congruency, *F*(1, 49) = 4.12, *p* = 0.048, *η*_*p*_^2^ = 0.08 indicating that participants made more errors on incongruent trials (1%) than congruent trials (0.6%). The Deaf did not differ with respect to normal-hearing, *F*(1, 49) = 0.41, *p* = 0.52, *η*_*p*_^2^ = 0.01. There was a significant interaction between prime location and congruency, *F*(1, 49) = 10.03, *p* = 0.003, *η*_*p*_^2^ = 0.17 indicating higher percentage of errors (*p* = 0.003) on incongruent trials (1.2%) than congruent trials (0.5%), but only for central primes. A significant interaction between SOA and congruency was also observed, *F* (1, 49) = 62.31, *p* < 0.001, *η*_*p*_^2^ = 0.56.

### Prime visibility

Signal detection measure *d*′ was calculated to assess prime visibility. The prime “1” was considered as Signal and “2” as Noise. Consequently, correct responses to the prime “1” qualified as Hits and incorrect responses to “2” qualified as False alarms (FA). Prime visibility was above chance level for each group: *d*′ = 1.4, *t* (1, 25) = 7.16, *p* < 0.001 for the Deaf and *d*′ = 0.87, *t* (1, 25) = 8.26, *p* < 0.001 for the normal-hearing participants.

To examine whether the two groups differed on the ability to discriminate between the two primes, repeated measures ANOVA was performed on *d*′ with prime location as a within-subjects factor and Group as a between-subjects factor. There was a main effect of group, *F*(1, 49) = 15.43, *p* < 0.001, *η*_*p*_^2^ = 0.24 indicating that the Deaf individuals (*d*′ = 1.4) could discriminate the primes better than the normal-hearing participants (*d*′ = 0.87). Central primes (*d*′ = 1.34) were discriminated better compared to peripheral primes (*d*′ = 0.94), as shown by a main effect of prime location, *F*(1, 49) = 12.4, *p* = 0.001, *η*_*p*_^2^ = 0.2. The interaction between prime location and group was significant, *F*(1, 49) = 32.45, *p* < 0.001, *η*_*p*_^2^ = 0.4 (Fig. 2D). Post-hoc analysis showed that Deaf participants were able to discriminate the primes better than normal-hearing only when they were presented at periphery (Deaf: 1.53 vs. normal-hearing: 0.34), but not at center (Deaf: 1.28 vs. normal-hearing: 1.39).

### Correlational analysis

To examine if visibility of the primes lead to the priming effects seen in the main task, linear regression analysis was performed with proportion of congruent choices as the dependent variable and *d*′ as the predictor variable separately for each prime location and each group. The analysis revealed a significant correlation between *d*′ and proportion of congruent choices for the Deaf group but only for primes presented at periphery, *r* = 0.47, *p* = 0.02 and not for those presented at center, *r* = 0.19, *p* = 0.36 (Fig. 2E and F). No significant correlations between *d*′ and proportion of congruent choices were observed for the normal-hearing group either at center, *r* = 0.22, *p* = 0.29 or at periphery, *r* = 0.18, *p* = 0.38 (Fig. 2G and H). Linear regression analysis was also performed with RT priming effect (in free- and forced-choice trials) as the dependent variable. Similar results were found for RT on free-choice trials (Supplementary Tables S5–S6).

## Discussion

Deaf participants showed a greater influence of the primes on choices and response times in both free- and forced-choice trials. Participants also made fewer errors on congruent trials, although no group difference was found in the percentage of errors. No effect of SOA was observed on any of the variables. Based on earlier evidence of central-peripheral asymmetry we expected the PCE to turn into NCE at long SOA (150 ms) only for central primes but not for peripheral primes in normal-hearing participants. Additionally, due to the peripheral advantages in the Deaf, we expected PCE to turn into NCE for both central and peripheral primes at long SOA. The failure to observe these could be due to several reasons. One could argue that the dissimilarity of our masks and the targets could be one factor since NCE is best observed when masks consist of task-relevant features^32^. Further, according to the activation followed by inhibition account, self-inhibition is triggered at longer SOAs since there is no longer any perceptual information triggered by the primes. Thus, NCE is commonly observed when the primes are completely invisible^8, 33^. Although this point is debatable, a negative correlation between prime visibility and size of NCE has been observed suggesting that the magnitude of self-inhibition goes up as visibility is reduced (see ref. 34 for a review). Thus, the lack of NCE in this experiment could also be attributed to the above chance level discrimination of the primes. Finally, an important difference between our study and previous studies that have observed central-peripheral asymmetry is the location of peripheral primes. While most such studies presented peripheral primes at less than 6 degrees away from the fixation, peripheral primes in our study were presented at 21 degrees (in line with previous studies on Deaf that have observed a peripheral advantage). This could be one of the reasons why PCE did not turn into NCE at longer SOAs for peripheral primes even for hearing impaired participants in spite of their heightened perceptual sensitivity.

Nonetheless. these data provide evidence for higher masked priming in the deaf. The visibility index also correlated significantly with priming effects for the peripheral primes in the Deaf. Thus, our results also suggest enhanced perceptual advantages for the Deaf at the periphery to masked stimuli. In the next experiment, we examined if the effect we observed had any attentional component.

## Experiment 2

In Experiment 1, we observed enhanced priming effects in the Deaf for both central and peripheral primes compared to the normal hearing participants. Studies have shown that varying prime/target location induces attentional demands on the participants^27, 35^. Existing evidence also suggests that peripheral visual processing advantage is found in the Deaf when selective attention is engaged either by a central task or if the peripheral stimuli are unpredictable (see [20] for a review). Accordingly, we observed higher priming effects at the periphery (also, at center) for the Deaf in Experiment 1 where selective attention was engaged at the fovea as a possible target location. In Experiment 2, we examined if the peripheral advantage in Deaf would disappear if the peripheral attentional engagement were less demanding. We tested this by also presenting the targets at the periphery (apart from the center). Thus, both the Deaf and normal-hearing participants’ attention was focussed to peripheral and central locations as possible target locations. Here, we expected the Deaf to show higher priming effects only at the center but not at the periphery. The SOA manipulation was maintained for the sake of consistency between Experiment 1 and 2 so that any differences in the effects in Experiment 2 could only be attributed to the manipulation of attentional engagement and not due to the differences in the mask-target SOA between the two experiments.

### Data analysis

Similar data trimming and analysis procedure were used as in Experiment 1.

#### Forced choice trials

Trials with no responses (0.9%) and trials with RT above or below 2 SD of the Mean RT of each participant (Deaf: 4.6% and normal-hearing: 4.2%) were discarded. Error trials were excluded from analysis (Deaf: 15.7% and normal-hearing: 4.1%). The overall range of the error percentage across the hearing-impaired participants was 0 – 48%. We found that the percentage of errors for the Deaf participants was fairly high because four of the participants gave inaccurate responses on more than 40% of the trials. The mean error percentage excluding these four participants was 6.4%. However, we decided to retain the data from these participants as the analysis after discarding them did not give different results (Supplementary Tables S1–S4). Errors were analysed by calculating *d*′ similar to Experiment 1.

#### Free choice trials

0.3% of the trials with no responses and 0.15% of trials with RT > 100 ms were discarded from analysis. The analysis procedure for the proportion of choices and RT was same as experiment 1.

## Results

### Free-choice trials

**Figure 3:**
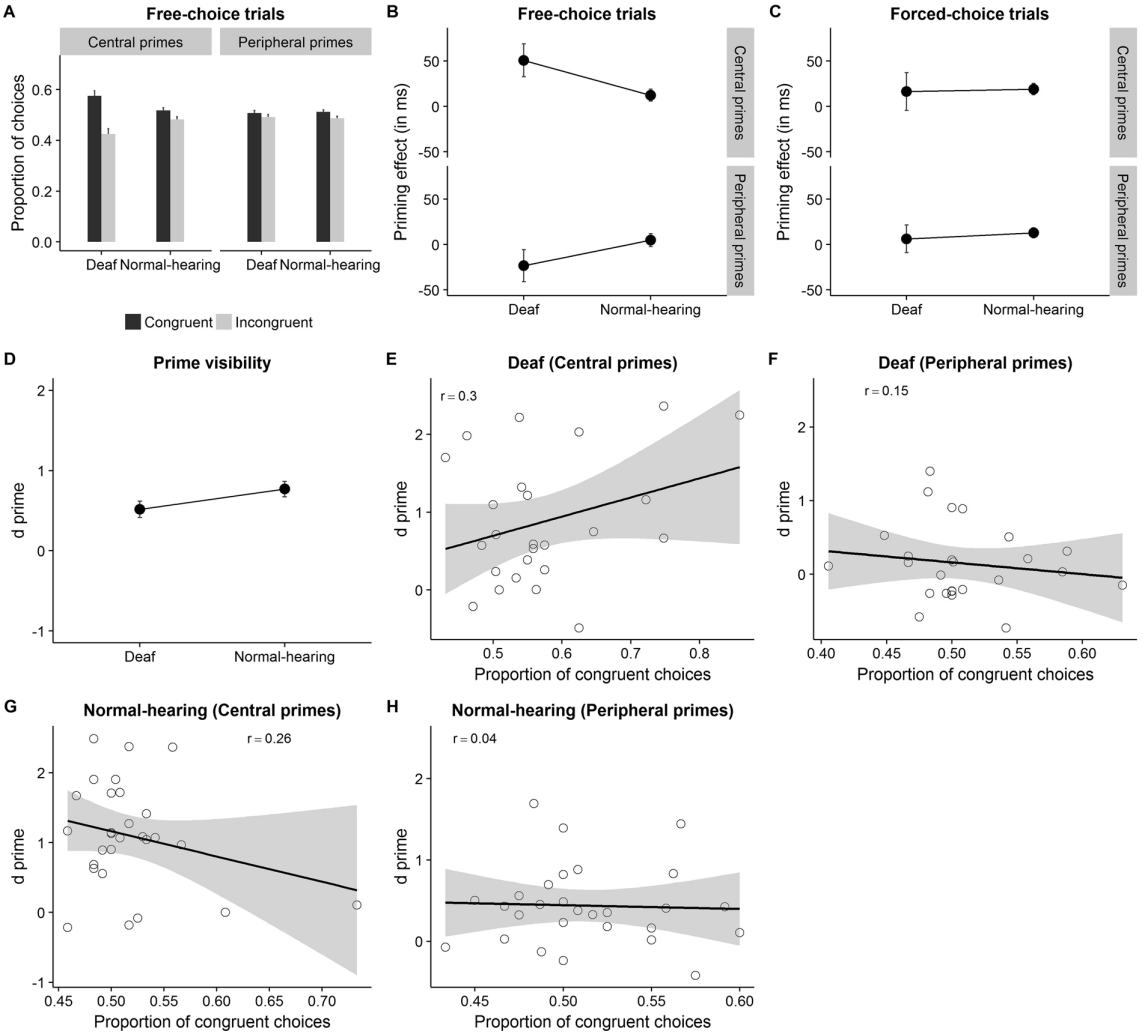
Experiment 2 results. (**A**) Proportion of congruent choices is higher for Deaf compared to normal-hearing, only for central primes. (**B**) Group difference on priming effect on free-choice trials, only for central primes (**C**) No group difference in priming effect on forced-choice trials (**D**) Marginally significant group difference in prime visibility index (**E**,**F**) Correlation plots between proportion of congruent choices and d prime. None of the correlations were significant.

Participants chose responses congruent with the prime (52.8%) more often than incongruent responses (47.2%), *t* (1, 52) = 4.26, *p* < 0.001 Repeated measures ANOVA was performed on *d*′ with prime location, SOA and congruency as within-subjects factors and group as a between-subjects factor (see Table 2 for descriptive statistics). There was a marginally significant effect of Group, *F*(1, 51) = 4.04, *p* = 0.05, *η*_*p*_^2^ = 0.07 indicating higher proportion of congruent choices in the Deaf (54.2%) compared to normal-hearing participants (51.5%, Fig. 3A). Individual t-tests showed that *d*′ differed significantly from zero for both Deaf (0.16) and normal-hearing participants (0.06), *t* (1, 24) = 4.64, *p* = 0.001and *t* (1, 27) = 2.45, *p* = 0.02, respectively. Proportion of congruent choices was also higher for central primes (54.7%) than peripheral primes (51%), as suggested by a main effect of prime location, *F*(1, 51) = 5.46, *p* = 0.02, *η*_*p*_^2^ = 0.09. There was a significant interaction between prime location and Group, *F*(1, 51) = 5.26, *p* = 0.026, *η*_*p*_^2^ = 0.09. Pairwise comparisons showed that the Deaf and normal-hearing group differed only at the centre (Deaf: 57.5% vs normal-hearing: 50.8%, *p* = 0.016) but not at periphery (Deaf: 51.8% vs normal-hearing: 51.3%, *p* = 0.73). The effect of SOA and its interaction with Group were both non significant, *F*(1, 51) = 1.58, *p* = 0.21, *η*_*p*_^2^ = 0.03 and *F*(1, 51) = 3.2, *p* = 0.08, *η*_*p*_^2^ = 0.06 respectively. None of the other effects were significant (*F*s < 1).

**Table 2:** Descriptive Statistics - Experiment 2.

			Central primes				Peripheral primes			
			0		150		0		150	
			C	IC	C	IC	C	IC	C	IC
**Free- choice**	**Choices**	**Deaf**	54 (9)	46 (9)	61 (13)	39 (13)	49 (17)	51 (17)	53 (18)	47 (18)
		**NH**	51 (6)	49 (6)	52 (8)	48 (8)	52 (11)	48 (11)	51 (12)	49 (12)
	**RT**	**Deaf**	604 (143)	642 (158)	494 (98)	560 (132)	701 (173)	646 (144)	529 (122)	548 (111)
		**NH**	610 (127)	621 (132)	610 (145)	624 (134)	631 (137)	639 (111)	605 (155)	607 (148)
**Forced choice**	**Error rate**	**Deaf**	1.3 (1.4)	1.8 (2.5)	1.8 (1.7)	2.8 (3.5)	2 (2.6)	2 (1.8)	1.9 (2.2)	2.2 (2.5)
		**NH**	0.4 (0.6)	0.5 (0.5)	0.4 (0.5)	0.6 (1.1)	0.6 (0.8)	0.4 (0.4)	0.5 (0.6)	0.7 (0.8)
	**RT**	**Deaf**	773 (180)	799 (201)	684 (188)	691 (168)	819 (191)	818 (197)	713 (208)	726 (196)
		**NH**	600 (98)	610 (103)	560 (97)	588 (93)	611 (91)	611 (80)	555 (99)	581 (89)

Note: Mean values with SD in brackets. C: Congruent, IC: Incongruent, NH: Normal-hearing, “0” and “150” refer to mask-target SOA in ms, Choices and error rates are given in percentage (%). RT is given in ms.

The ANOVA on response times in free-choice trials revealed a marginally significant effect of congruency, *F* (1, 51) = 2.89, *p* = 0.09, *η*_*p*_^2^ = 0.05. Congruent trials (597.9 ms) elicited faster responses than incongruent trials (610.78 ms). ANOVA was then performed on RT priming effect (incongruent RT – congruent RT) which revealed no effect of group on priming effect, *F*(1, 51) = 0.11, *p* = 0.74, *η*_*p*_^2^ = 0.002. Prime location had a significant effect, *F*(1, 51) = 15.94, *p* < 0.001, *η*_*p*_^2^ = 0.24. The RT priming effect for central primes (31.55 ms) was higher compared to peripheral primes (−9.25 ms). There was a significant interaction between prime location and group, *F*(1, 51) = 10.65, *p* = 0.002, *η*_*p*_^2^ = 0.17 (Fig. 3B). Pairwise comparisons showed that the two groups differed in priming effect only when primes were presented at the center (Deaf: 50.79 ms vs normal-hearing: 12.31 ms, *p* = 0.04) but not at periphery (Deaf: −23.35 ms vs normal-hearing: 4.85 ms, *p* = 0.13). There was a significant effect of SOA, *F*(1, 51) = 5.08, *p* = 0.03, *η* ^2^ = 0.09 indicating higher priming effects for 150 ms SOA (27.48 ms) compared to 0 ms SOA (−5.18 ms). The interaction between SOA and group was significant, *F*(1, 51) = 5.45, *p* = 0.02, *η*_*p*_^2^ = 0.1. Pairwise comparisons showed that priming effect was higher (marginally significant, *p* = 0.05) for the Deaf (46.96 ms) compared to normal-hearing (7.99 ms) participants at 150 ms SOA but not at 0 ms SOA (*p* = 0.2) None of the other effects were significant (Fs < 3).

### Forced-choice trials

Repeated measures ANOVA was performed on RT with prime location (central, peripheral), SOA (0 ms, 150 ms) and congruency (congruent, incongruent) as within-subjects factors. Group (Deaf, normal-hearing) was considered as a between-subjects factor. Responses on congruent trials (664.61 ms) were faster compared to incongruent trials (678.24 ms), as shown by a marginally significant effect of congruency, *F* (1, 51) = 3.87, *p* = 0.05, *η*_*p*_^2^ = 0.07. To examine whether this priming effect was modulated by the other factors, repeated measures ANOVA was performed on RT priming effect (RT on incongruent trials – RT on congruent trials) with prime location, SOA and group as factors. There was no group difference in the RT priming effect, *F*(1, 51) = 0.11, *p* = 0.74, *η*_*p*_^2^ = 0.002 (Deaf: 11.35 ms vs normal-hearing: 15.92 ms, Fig. 3C). Prime location had no significant effect, *F*(1, 51) = 4.82, *p* = 0.49, *η*_*p*_^2^ = 0.009. The interaction between prime location and Group, *F*(1, 51) = 0.03, *p* = 0.87, *η*_*p*_^2^ = 0.001 was not significant either. The main effect of SOA was not significant (F < 1). None of the other interactions were statistically significant (Fs < 2).

ANOVA on *d*′ values for error data revealed a significant effect of group, *F*(1, 51) = 14.01, *p* < 0.001, *η*_*p*_^2^ = 0.21. Deaf participants made larger number errors (2%) than normal-hearing participants (0.5%). Participants were in general more error-prone on incongruent trials (1.4%) than congruent trials (1.1%), *F*(1, 51) = 9.43, *p* = 0.003, *η*_*p*_^2^ = 0.16. The interaction between congruency and group was not significant, *F*(1, 51) = 1.11, *p* = 0.3, *η*_*p*_^2^ = 0.02. The interaction of congruency with prime location was not significant either, *F*(1, 51) = 2.45, *p* = 0.12, *η*_*p*_^2^ = 0.05. There was a significant effect of SOA*, F*(1, 51) = 8.16, *p* = 0.006, *η*_*p*_^2^ = 0.14 indicating higher percentage of errors for trials with 150 ms SOA (1.3%) compared to 0 ms SOA (1.1%).

### Prime visibility

*d*′ was calculated using a procedure similar to that of Experiment 1. Prime discrimination was above chance level for each group, *d*′ = 0.52, *t* (1, 24) = 5.16, *p* < 0.001 for the Deaf, *d*′ = 0.77, *t* (1, 27) = 8.76, *p* < 0.001 for the normal-hearing participants. ANOVA was performed on *d*′ with prime location and group as factors. The analysis revealed a marginally significant effect of Group, *F*(1, 51) = 3.32, *p* = 0.07, *η*_*p*_^2^ = 0.06 (Fig. 3D). Normal-hearing participants were better at discriminating primes (*d*′ = 0.52) compared to the Deaf participants (*d*′ = 0.77). Central primes (*d*′ = 0.99) were discriminated better than peripheral primes (*d*′ = 0.29), *F*(1, 51) = 35.36, *p* < 0.001, *η*_*p*_^2^ = 0.41. Interestingly, there was also a main effect of SOA, *F*(1, 51) = 13.43, *p* = 0.001, *η*_*p*_^2^ = 0.21. Primes presented with 150 ms SOA were discriminated better (*d*′ = 0.84) compared to those with 0 ms SOA (*d*′ = 0.44). None of the other effects were statistically significant.

### Correlational analysis

Linear regression analysis was performed with proportion of congruent choices as dependent variable and *d*′ as the predictor separately for each group at both centre and periphery. No significant correlation was observed between the two variables for the Deaf group when the primes were presented at centre, *r* = 0.31, *p* = 0.14 as well as at periphery, *r* = 0.15, *p* = 0.48 (Fig. 3E and F). Similarly, for the normal-hearing participants, no significant correlations between prime visibility and proportion of congruent choices were observed for central primes, *r* = 0.26, *p* = 0.19 and peripheral primes, *r* = 0.04, *p* = 0.84 (Fig. 3G and H). Linear regression analysis with RT priming effect on free- and forced-choice trials as the dependent variable is reported in Supplementary Tables S7–S8. No significant effects were observed.

## Discussion

In this experiment, we observed that the two groups did not differ on the degree of priming for peripheral primes when attentional demand was higher. Deaf showed higher priming effects on response times and proportion of choices on free trials but only for the central primes. No group differences were observed on forced-choice priming effects. Similar to the results of Experiment 1, both groups could discriminate the primes at higher than chance level. Normal-hearing participants were marginally better at discriminating primes compared to the Deaf. However, no significant correlations were observed between priming effect and prime discrimination index for either of the groups at either of the locations. The results suggest that peripheral advantage in the deaf emerges only under certain conditions, mostly when their attention is spread over a large area.

## General discussion

Our results provide strong evidence of enhanced masked priming in the deaf. We have shown that the peripheral advantage is seen when the attentional demands are higher (Experiment 1). These results extend previous findings and show that even processing of masked stimuli is modified as a result of neuroplasticity. For the first time, we have shown that sensory deprivation in the deaf not only facilitates processing of perceivable visual events but also visual cues that are hardly visible. Importantly, such cues could influence choices of voluntary actions and facilitate actions themselves. The results thus extend earlier findings that have shown superior peripheral processing in the deaf (e.g. refs 11, 25). They also extend results obtained with the normal-hearing population that have shown the influence of subliminal or masked stimuli on conscious decision making in visuomotor actions^36, 37^.

Enhanced processing of peripheral primes in the Deaf was observed only in Experiment 1 since participants’ selective attention was engaged at centre due to the repeated appearance of the target. This difference disappeared in Experiment 2 where the targets could appear both at the periphery and at centre. This made the task attentionally less demanding at the periphery. This is in line with previous studies where no peripheral differences between Deaf and normal-hearing have been observed when the tasks were not attentionally demanding^15^ (also see ref. 38). Our results suggest that sensitivity to masked primes is modulated by the redistribution of attentional resources between the centre and the periphery^25, 39^. Further, in both the experiments, we observed an enhanced processing of central primes in the Deaf. Enhanced processing of foveal stimuli has not been consistently found in previous studies^38–40^. Thus, whether plasticity-related changes in Deaf also result in higher processing of stimuli presented at fovea is still an open question^41^.

We expected the time course of priming effects to be influenced differently in Deaf and normal-hearing participants. The mask-target SOA had no significant influence on the prime’s influence in both the experiments. Further, we did not observe the central-peripheral asymmetry in normal-hearing individuals seen in previous studies (e.g. ref. 27). The possible reasons for this discrepancy have been discussed. However, it ‘s hard to draw firm conclusions since we only manipulated two levels of SOA. Lingnau and Vorberg^42^ examined the time course of response priming effects by systematically varying mask-target SOA, stimulus size and eccentricity. They observed that several factors could influence the onset of response inhibition and that NCE might be missed by measuring priming effects at only one or two SOAs. Thus, future work aiming to examine central/peripheral priming effects in deaf and normal-hearing participants should systematically vary both stimulus eccentricity and mask-target SOA thereby shedding more light on the time course of these effects.

A possible limitation of our study is that the prime visibility was not at chance level (but see refs 34, 43 for similar results). However, the prime discrimination measure is an overestimation of the actual visibility of the primes in the main experiment since the participants are explicitly made aware of the primes in the prime discrimination test and are asked to focus on detecting the primes. Further, we did not observe any correlations between prime discriminability index and the priming effects in masked priming tasks, except at periphery in Experiment 1. Budnik *et al*.^44^ had participants perform a masked priming task with Gabor patches as primes presented at both centre and periphery. The main task consisted of responding to the orientation of a visible Gabor patch. A clear difference between central and peripheral priming effects was observed even though prime visibility was controlled for (by equating the discrimination performance at centre and periphery using a staircase procedure) suggesting that separate mechanisms are likely responsible for the unconscious activations triggered by the primes and the visibility of the primes. Thus, our results support many previous studies which show that the influence of the masked primes on subsequent behaviour is independent of the level of awareness of the primes. (e.g. refs 17, 45, 46). We acknowledge that we can’t be confident that these effects will still be observed with stimuli that are entirely invisible and unconscious. Recently many have recommended newer methods to ensure and evaluate invisibility of the primes^47, 48^. Future work examining the role of auditory deprivation on unconscious processing should consider these newer methods and employ stronger procedures to ensure the invisibility of the primes.

In sum, we have shown that sensory deprivation in the deaf can enhance the attentional and visual systems to the extent that these systems become highly sensitive to even information that is hardly visible. Such information not only influenced the choice of free actions but also the actions themselves. Additionally, these effects in the deaf are modulated by the redistribution of the attentional resources between the fovea and periphery. However, from these results, one cannot say if the prime is influencing the action itself or mental states related to the action. These questions need to be further examined from the point of view of brain plasticity and individual differences.

## Method

### Experiment 1

#### Participants

Twenty-eight hearing-impaired and twenty-seven normal-hearing participants initially took part in the main priming study. Prime visibility data from three hearing impaired individuals and one normal-hearing participant was not available (due to technical problems, failure of the participant in understanding instructions, etc.). Thus, data from twenty-five hearing impaired individuals (4 female, Mean age = 24.4 years, SD = 4.3) and twenty-six normal-hearing individuals (10 female, Mean age = 24.7 years, SD = 4.8) were finally considered for the study. The deaf participants were from Deaf Enabled Education Centre (refer Table 3 for details on deaf participants). All deaf participants were congenitally deaf, born to hearing parents and suffered from profound sensorineural hearing loss. They received education in special schools for the deaf in which the primary medium of communication was Indian Sign Language. They reported having acquired sign language at an average age of 9.4 years and reported high proficiency in sign language use (Mean self-rating score: 3; see ref. 18 for details of this scale). Participants with normal hearing were all students at the University of Hyderabad. All participants reported normal or corrected-to-normal vision and gave informed consent for their participation in the study. Instructions to the hearing impaired participants were given by one of the co-authors who is a speech therapist with good proficiency in sign language. The study protocol was approved by the Institutional Ethics Committee (IEC) at University of Hyderabad. The methods were in accordance with the guidelines and regulations of IEC. None of the participants had been diagnosed with any psychiatric/neurological condition.

**Table 3:** Details of Deaf participants (Experiment 1 and 2).

Experiment 1			Experiment 2		
Subject	Duration of use of hearing devices (in years)	Age of acquisition of sign language (in years)	Subject	Duration of use of hearing devices (in years)	Age of acquisition of sign language (in years)
D1	10	8	D1	5	13
D2	12	10	D2	0	6
D3	10	10	D3	0	15
D4	10	9	D4	20	6
D5	19	14	D5	12	3
D6	7	15	D6	13	6
D7	10	10	D7	12	10
D8	8	15	D8	6	5
D9	5	8	D9	8	9
D10	7	6	D10	9	13
D11	10	8	D11	0	5
D12	5	5	D12	22	14
D13	7	6	D13	10	7
D14	8	8	D14	18	6
D15	8	8	D15	3	22
D16	8	9	D16	6	5
D17	10	7	D17	0	16
D18	6	9	D18	6	17
D19	10	16	D19	10	9
D20	12	15	D20	10	12
D21	10	12	D21	6	6
D22	10	14	D22	0	8
D23	12	15	D23	8	8
D24	12	13	D24	0	5
D25	8	12	D25	0	8

### Stimuli and Procedure

Stimuli were designed and presented using the SR research experiment builder (SR Research, Ontario, Canada) on an LCD monitor with resolution 1024 * 768 pixels and refresh rate of 60 Hz. Participants were seated at a distance of 60 cm from the monitor. The stimuli were presented in black against a white background. Every trial started with a fixation cross at the centre of the screen for 1000 ms (See Fig. 1A). Following the fixation cross, a prime was presented for 33 ms. Next, a line mask (“######”, Times new roman, pt. 26) was presented for 50 ms followed by a blank screen of variable duration (0 ms/150 ms). The primes constituted of the digits “1” or “2” (Times new roman, pt. 26) and were located either at the centre or the periphery (21-degree eccentricity to the left/right of the fixation). There were two types of trials, “Free-choice” and “Forced-choice” which were presented randomly. On the forced-choice trials, the blank screen was followed by the presentation of the target (“1” or “2”) at the centre of the screen. Participants were asked to press “A” for “1” and “L” on the keyboard for “2”. On free-choice trials, participants were presented with “0” at the centre after the blank screen and were asked to freely choose their response between “A” and “L”. Participants were asked to “choose freely and spontaneously” without following a particular pattern. Participants were given a maximum of 3000 ms to respond to the target on forced-choice trials or to choose a response on free-choice trials. The mapping between the target and the response keys on the keyboard was counterbalanced across participants.

After the main experiment, participants performed a prime visibility task. Primes (1 or 2) were presented for 33 ms followed by a mask for 50 ms. After a variable duration (0, 150 ms), participants were presented with a screen displaying “1 or 2?” for 5000 ms. Participants were asked to press the key corresponding to the number they detected (that is, 1 or 2 on the keyboard).

### Design

There was a total of 400 trials divided into two blocks of 200 trials each. Each block consisted of 120 free-choice trials and 80 forced-choice trials. The 120 free-choice trials in each block were divided into 60 trials with central primes and 60 with peripheral primes. Each of these 60 trials was further divided into 30 trials with 0 ms SOA and 30 trials with 150 ms SOA. The 80 forced-choice trials in each block were also divided equally between the eight types of trials: prime location (2) * SOA (2) * congruency (2) with ten trials in each condition. A forced-choice trial was defined as “congruent” when the prime matched with the target (e.g. prime – 1, target - 1) and as “incongruent” when there was a mismatch (e.g. prime – 2, target - 1). On free-choice trials, if the participant chose the response indicated by the prime, it was designated as a “congruent” trial (e.g. when participants pressed “A” when the prime was 1) and vice versa. The trials in each block were presented in a random sequence. A break of two minutes was given after one block. Each participant was given twenty practice trials before the start of the experiment.

## Experiment 2

### Participants

Thirty hearing-impaired and thirty normal hearing individuals took part in the main priming study out of which prime visibility data from five hearing-impaired and two normal-hearing participants was not available. Hence, data from twenty-five hearing-impaired individuals (23 male, Mean age = 25.2 years, SD = 3.6) and twenty-eight normal-hearing individuals (22 male, Mean age = 24.9 years, SD = 3.9) was eventually considered for all analysis. The characteristics of the Deaf participants were similar to those who participated in Experiment 1 (See Table 3). The average age of acquisition of sign language was 7.4 years and mean self-rating score on sign language proficiency was 2.3. None of the participants who took part in Experiment 1 were recruited for this Experiment. The experimental protocols were approved by the Institutional Ethics Committee (IEC) at University of Hyderabad. The methods were carried out in accordance with the regulations of IEC. All participants gave informed consent for their participation in the experiment.

## Stimuli and Procedure

The procedure was the same as in Experiment 1 except as noted below. In Experiment 1, primes were presented either at the centre or the periphery whereas targets were always presented in the centre. In this Experiment, the prime and the target location always matched (Fig. 1B). Thus, peripheral primes were followed by targets at the periphery and central primes were followed by targets at the centre. The trial structure and the design of the experiment remained same.

The sequence of events in the prime visibility task was similar to the main Experiment. Similar to Experiment 1 prime visiblity test, participants were asked to judge if the prime was “1”or “2” and respond through the keyboard within 5000 ms.

### Data availability

The datasets generated during and/or analysed during the current study are available in the OSF repository, https://osf.io/kdqau/?view_only=f45d0d11563c4698b95a384220eb8177.

## Electronic supplementary material

Supplementary information
