# Supplementary material for: Cross-modal plasticity in the deaf enhances processing of masked stimuli in the visual modality
Source: Sci Rep. 2017 Aug 15;7:8158. doi: 10.1038/s41598-017-08616-4 (PMC5558002; doi:10.1038/s41598-017-08616-4)
Supplement: Supplementary file 1 — Supplementary information [file 41598_2017_8616_MOESM1_ESM.doc]

**Supplementary material**

**Cross-modal plasticity in the deaf enhances processing of masked stimuli in the visual modality**

Seema Prasad^1^, Gouri Shanker Patil^2^, Ramesh Kumar Mishra^1^

^1^Center for Neural and Cognitive Sciences, University of Hyderabad

^2^Ali Yavar Jung National Institute for the Hearing Handicapped, Secunderabad, India

Corresponding author:

Ramesh Kumar Mishra

Center for Neural and Cognitive Sciences, Science Complex

University of Hyderabad,

Hyderabad, Telangana 500046

Ph No: 09451872007

Email id: rkmishra@uohyd.ac.in

The analysis of the priming task data was repeated after excluding the four participants whose error percentage was greater than 40 % on forced-choice trials. The analysis procedure was the same as mentioned in the main text. The important main effects and interactions are given in the tables below:

**Experiment 2 (n = 49)**

Supplementary Table S1. ANOVA on Percentage of congruent choices: Free-choice trials

| Factor | F statistics | Means |
| --- | --- | --- |
| Group | *F*(1, 47) = 3.08, *p* = 0.08^†^ , *η_p_^2^ =* 0.06 | Deaf: 53.9 % (SE = 4)  Normal-hearing: 51.5 % (SE = 0.4) |
| Location | *F*(1, 47) = 9.13, *p* = 0.004* , *η_p_^2^ =* 0.16 | Centre: 54.47 % (SE = 2)  Periphery: 50.9 % (SE = 3) |
| Group * Location | *F*(1, 47) = 6.64, *p* = 0.01* , *η_p_^2^ =* 0.12 | Centre  Deaf: 57.15 % (SE = 4)  Normal-hearing: 51.8 % (SE = 0.6) |
|  |  | Periphery  Deaf: 50.6 % (SE = 9)  Normal-hearing: 51.2 % (SE = 0.9) |

* significant, ^†^ marginally significant

Supplementary Table S2. ANOVA on priming effect (Incongruent RT – Congruent RT): Free-choice trials

| Factor | F statistics | Means |
| --- | --- | --- |
| Group | *F*(1, 47) = 1.43, *p* = 0.24, *η_p_^2^ =* 0.03 | - |
| Location | *F*(1, 47) = 16.55, *p* , 0.001* , *η_p_^2^ =* 0.26 | Centre: 36 ms (SE = 8)  Periphery: = -3 ms (SE = 8) |
| Group * Location | *F*(1, 47) = 10.78, *p* , 0.002* , *η_p_^2^ =* 0.19 | Centre  Deaf: 59 ms (SE = 12)  Normal-hearing: 12 ms (SE = 10) |
|  |  | Periphery  Deaf: -10 ms(SE = 13)  Normal-hearing: 5 ms (SE = 11) |

* significant,

Supplementary Table S3. ANOVA on priming effect (Incongruent RT – Congruent RT): Forced-choice trials

| Factor | F statistics | Means |
| --- | --- | --- |
| Group | *F*(1, 47) = 1.7, *p* = 0.2, *η_p_^2^ =* 0.03 | - |
| Location | *F*(1, 47) < 0.001, *p* = 0.9, *η_p_^2^ <* 0.001 | - |
| Group * Location | *F*(1, 47) = 0.58, *p* = 0.5, *η_p_^2^ =* 0.01 | - |

Supplementary Table S4. Error percentage: Forced-choice trials.

| Factor | F statistics | Means |
| --- | --- | --- |
| Congruency | *F*(1, 47) = 2.6, *p* = 0.11, *η_p_^2^ = 0.05* | - |
| Group | *F*(1, 47) = 10.9, *p* = 0.002*, *η_p_^2^ = 0.19* | Deaf: 1.2 % (SE = 0.2)  Normal-hearing: 0.5 % (SE = 0.1) |
| Group * Congruency | *F*(1, 47) < 1 | - |

* significant

**Correlational analysis**

Linear regression analysis was performed with congruency effect as dependent variable and *d'* as the predictor separately for each group at both centre and periphery. Results are tabulated below:

**Experiment 1.**

Supplementary Table S5. Priming effect for responses on forced-choice trials vs d prime

| Group | Prime location | *r* | *P* |
| --- | --- | --- | --- |
| Deaf | Centre | 0.22 | 0.29 |
|  | Periphery | 0.32 | 0.12 |
| Normal | Centre | 0.12 | 0.56 |
|  | Periphery | 0.18 | 0.38 |

Supplementary Table S6. Priming effect for responses on free trials vs d prime

| Group | Prime location | *r* | *P* |
| --- | --- | --- | --- |
| Deaf | Centre | 0.07 | 0.72 |
|  | Periphery | 0.49 | 0.01* |
| Normal | Centre | 0.08 | 0.7 |
|  | Periphery | 0.28 | 0.16 |

*significant

**Experiment 2**

Supplementary Table S7. Priming effect for responses on forced-choice trials vs d prime

| Group | Prime location | *r* | *P* |
| --- | --- | --- | --- |
| Deaf | Centre | 0.26 | 0.2 |
|  | Periphery | 0.21 | 0.31 |
| Normal | Centre | 0.17 | 0.38 |
|  | Periphery | 0.17 | 0.39 |

Supplementary Table S8. Priming effect for responses on free-choice trials vs d prime

| Group | Prime location | *r* | *p* |
| --- | --- | --- | --- |
| Deaf | Centre | 0.34 | 0.1 |
|  | Periphery | 0.004 | 0.9 |
| Normal | Centre | 0.12 | 0.56 |
|  | Periphery | 0.37 | 0.05^†^ |

^†^ marginally significant
